# Poplar PdMYB221 is involved in the direct and indirect regulation of secondary wall biosynthesis during wood formation

**DOI:** 10.1038/srep12240

**Published:** 2015-07-16

**Authors:** Xianfeng Tang, Yamei Zhuang, Guang Qi, Dian Wang, Huanhuan Liu, Kairong Wang, Guohua Chai, Gongke Zhou

**Affiliations:** 1 Key Laboratory of Biofuels, Chinese Academy of Sciences, Shandong Provincial Key Laboratory of Energy Genetics, Qingdao Institute of Bioenergy and Bioprocess Technology, Chinese Academy of Sciences, Qingdao, 266101, China; 2 Qingdao Engineering Research Center for Rural Environment, College of Resources and Environment, Qingdao Agricultural University, Qingdao, 266109, China

## Abstract

Wood is formed by the successive addition of secondary xylem, which consists of cells with a conspicuously thickened secondary wall composed mainly of cellulose, xylan and lignin. Currently, few transcription factors involved in the direct regulation of secondary wall biosynthesis have been characterized in tree species. Here, we show that PdMYB221, a poplar ortholog of the Arabidopsis R2R3-MYB transcription factor AtMYB4, directly regulates secondary wall biosynthesis during wood formation. *PdMYB221* is predominantly expressed in cells of developing wood, and the protein it encodes localizes to the nucleus and acts as a transcriptional repressor. Ectopic expression of *PdMYB221* resulted in reduced cell wall thicknesses of fibers and vessels in Arabidopsis inflorescence stems. The amounts of cellulose, xylose, and lignin were decreased and the expression of key genes synthesizing the three components was suppressed in *PdMYB221* overexpression plants. Transcriptional activation assays showed that PdMYB221 repressed the promoters of poplar *PdCESA7/8*, *PdGT47C*, *PdCOMT2* and *PdCCR1*. Electrophoretic mobility shift assays revealed that PdMYB221 bound directly to the *PdCESA8*, *PdGT47C*, and *PdCOMT2* promoters. Together, our results suggest that PdMYB221 may be involved in the negative regulation of secondary wall formation through the direct and indirect suppression of the gene expression of secondary wall biosynthesis.

Wood, a dominant terrestrial biomass, is used for myriad applications such as building construction, paper making, pulping, furniture, and as a promising feedstock for biofuel. Wood is formed by the successive addition of secondary xylem, which originates from the vascular cambium and consists mainly of fibers and vessel elements^1^. Secondary xylem, in both herbaceous and woody plants, consists of cells with a conspicuously thickened secondary wall that develops beneath the primary cell wall and is predominantly composed of cellulose, xylan and lignin. Because of massive economic importance of wood, understanding how secondary wall biosynthesis during wood formation is regulated could potentially provide genetic tools for engineering wood components.

Recent studies in tree species have demonstrated that secondary wall biosynthesis during wood development is mediated by a multileveled network that coordinates the expression of the hundreds of genes in this process^2^,^3^. A group of wood-associated NAC domain transcription factors (WNDs), such as PtrWND2B and PtrWND6B, have been shown to be the top master switches regulating the expression of a number of transcription factors, which ultimately lead to the biosynthesis of secondary walls in poplar^3^,^4^,^5^. Several R2R3-MYB transcription factors have been demonstrated to function as second-level master switches regulating secondary wall formation. Of them, PtrMYB2, PtrMYB3, PtrMYB20 and PtrMYB21 are direct targets of PtrWNDs and share similar functions to their Arabidopsis orthologs MYB46 and MYB83^6^,^7^. These PtrMYBs positively regulate secondary wall formation and have the abilities of activating secondary wall biosynthetic pathways. Interestingly, poplar PtrMYB152, an ortholog of Arabidopsis MYB43, is not induced by *PtrWND2B* overexpression but functions in the regulation of secondary wall biosynthesis, suggesting the presence of a PtrWND2B-independent pathway governing secondary wall biosynthesis^8^. Our recent study shows that PdC3H17 and PdC3H18 are direct targets of PdMYB3 and PdMYB21 and function as positive regulators in the differentiation of vascular cambium and secondary wall thickening in poplar^9^. Overexpression of *PdC3H17* or *PdC3H18* in poplar activates the expression of genes involved in the biosynthesis of cellulose, xylan and lignin.

Compared with many indirect regulators identified, few genes involved in the direct regulation of secondary wall biosynthesis during wood formation have been characterized in tree species. Only three studies in Arabidopsis and switchgrass to date show that transcription factors can directly control secondary wall biosynthesis. Arabidopsis MYB46 functions as second-level master switch that regulates many secondary wall-associated genes^10^. It has recently been shown that MYB46 directly activates the expression of three cellulose synthetic genes *CESA4*, *CESA7* and *CESA8* by binding to the M46RE sequences of their promoters^11^. Arabidopsis MYB58, MYB63 and switchgrass PvMYB4, three lignin-specific regulators, have been shown to have the abilities of binding to the AC elements of the promoters of some lignin synthetic genes to directly activate the expression of these genes^12^,^13^,^14^. Bioinformatic analysis of the promoters of all lignin biosynthetic genes in Arabidopsis indicated that such AC elements are present in the majority of the genes except for the promoters of *ferulate 5-hydroxylase* (*F5H*)^15^. It is supposed that the AC elements may be specific for the regulation of lignin biosynthetic genes^15^,^16^. However, little is known whether the AC elements are involved in the regulation of gene expression of cellulose and xylan biosynthesis.

Arabidopsis MYB4, a transcriptional repressor, has previously been shown to negatively regulate the accumulation of the UV-protectant compound sinapoylmalate by repressing the expression of the gene encoding the phenylpropanoid enzyme cinnamate 4-hydroxylase^17^. Recently, several studies demonstrate that MYB4 may be involved in the regulation of secondary wall biosynthesis. It can not only be activated by MYB46, a second-level master regulator of secondary wall biosynthesis^18^, but also suppress the expression of the first-level master regulator SND1/NST3 through a feedback loop^19^,^20^. In addition, PvMYB4, the switchgrass ortholog of Arabidopsis MYB4, has been shown to be a negative regulator of lignin biosynthesis^14^.

In this study, we investigated the role of PdMYB221, a poplar ortholog of Arabidopsis MYB4, in secondary wall formation, and determined its targets involved in cellulose, xylan and lignin biosynthesis. We showed that ectopic expression of *PdMYB221* in Arabidopsis led to a reduction in secondary cell wall thicknesses of fibers and vessels. The amounts of cellulose, xylan, and lignin were decreased and the expression of key genes synthesizing the three components was suppressed in transgenic plants. Furthermore, we demonstrated that PdMYB221 suppressed the expression of the poplar *PdCESA7/8*, *PdGT47C*, *PdCOMT2* or *PdCCR1* promoters and had the abilities of binding to the AC elements of the *PdCESA8*, *PdGT47C*, and *PdCOMT2* promoters. Our results suggest that poplar *PdMYB221* may be involved in the negative regulation of secondary wall biosynthesis through the direct and indirect suppression of the expression of secondary wall biosynthetic genes.

## Results

### PdMYB221 is highly conserved in a range of plant species

**Figure 1:**
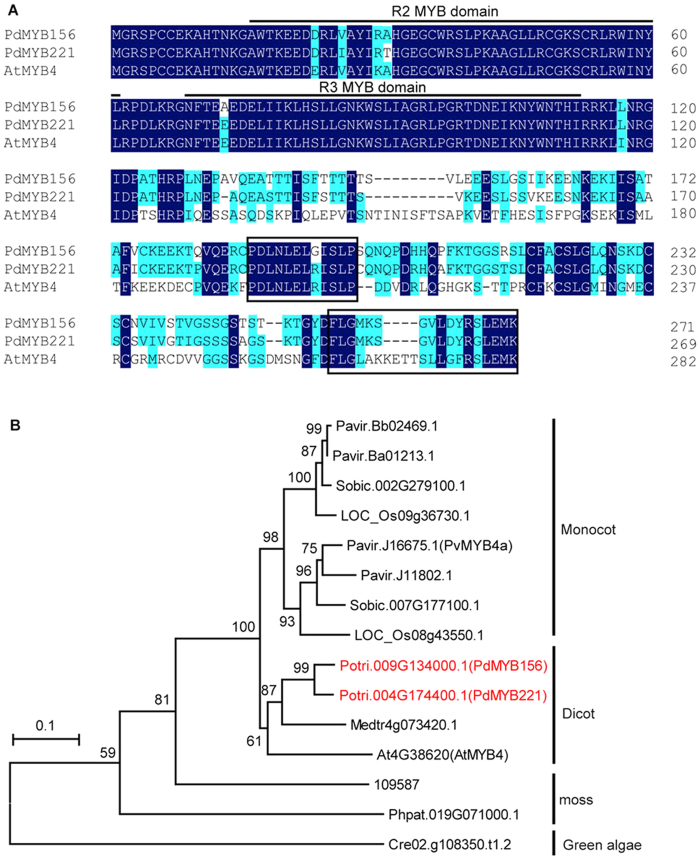
PdMYB221 is highly conserved in a broad range of plant species. (**A**) ClustalW alignment of the amino acid sequences of poplar PdMYB221, its paralog PdMYB156, and Arabidopsis AtMYB4. The R2, R3 MYB domains are underlined. The boxed sequences are the potential repression motifs. (**B**) Phylogenetic analysis of PdMYB221, PdMYB156, and thirteen orthologs from eight species. LOC_Os09g36730.1 and LOC_Os08g43550.1, *Oryza sativa*; PvMYB4a, Pavir.Bb02469.1, Pavir.Ba01213.1, and Pavir.J11802.1, *Panicum virgatum*; Medtr4g073420.1, *Medicago truncatula*; Sobic.007G177100.1 and Sobic.002G279100.1, *Sorghum bicolor*; Cre02.g108350.t1.2, *Chlamydomonas reinhardtii*; Phpat.019G071000.1, *Physcomitrell patens*; 109587, *Selaginella moellendorffii*. The unrooted tree was inferred by MEGA 4.0 with neighbor-joining method after the alignment of the full-length amino acid sequences. The number beside the branches represents bootstrap value based on 1,000 replications.

The *Populus trichocarpa* genome contains 192 R2R3-MYB family members, which form 81gene pairs^21^,^22^. Of them, PdMYB156 and PdMYB221 are a pair of paralogous genes, with 88% sequence identity over their full lengths and 97% identity over their R2R3-MYB domains. Both proteins contain the potential repression motifs and are the orthologs of Arabidopsis AtMYB4 (Fig. 1A), consistent with the previous phylogenetic analysis^21^. Thirteen orthologs of PdMYB156 and PdMYB221 were identified in eight representative plant species, including green algae, moss, spike moss, Arabidopsis, rice, sorghum, alfalfa and switchgrass (Fig. 1B). Phylogenetic analysis revealed that the fifteen R2R3-MYB proteins including PdMYB156/221 have high bootstrap percentage values and are therefore relatively conserved. By contrast, PdMYB156 and PdMYB221 fell into the group containing Arabidopsis AtMYB4 and alfalfa Medtr4g073420.1, suggesting that these proteins are more evolutionary conserved within dicots than in monocots, moss or green algae.

### *PdMYB221* and its paralog *PdMYB156* are predominantly expressed in fibers and vessels of poplar stems

**Figure 2:**
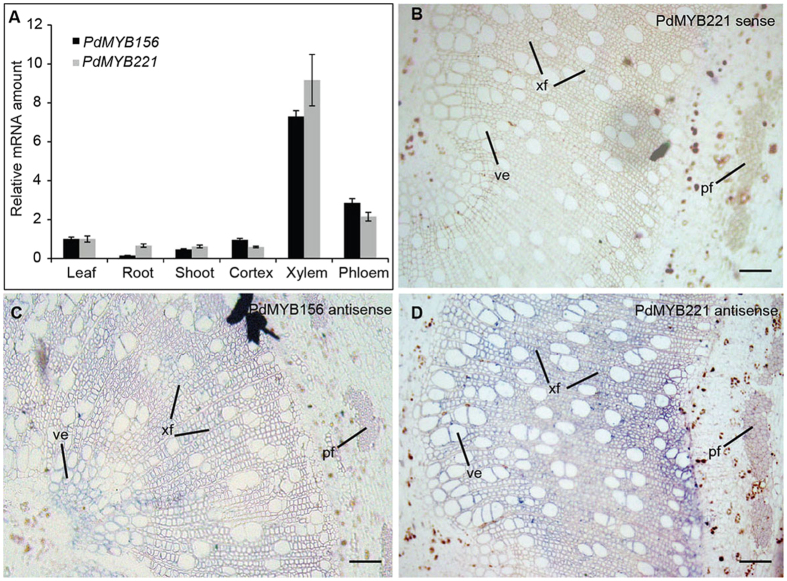
Expression patterns of *PdMYB221* and its paralog *PdMYB156*. (**A**) Relative expression levels of *PdMYB221* and *PdMYB156* in six poplar tissues by qRT-PCR analysis. *PdUBQ10* was used as a reference and expression level of *PdMYB156/221* in leaves was set as 1. Error bars represent SD of three biological replicates. (**B–D**) *In situ* mRNA localization of *PdMYB221* and *PdMYB156* in poplar stem sections. The hybridization signals are shown in purple. ve, vessel; xf, xylary fiber; pf, phloem fiber. Bars, 25 μm.

qRT-PCR was first performed to investigate expression patterns of *PdMYB156* and *PdMYB221* in six tissues of poplar. The results showed that both genes were expressed in all the tissues tested, with highest expression in xylem and phloem of the stems (Fig. 2A). *In situ* hybridization analysis of stem sections indicated that *PdMYB156* and *PdMYB221* were highly expressed in xylary fiber, vessel, and phloem fiber cells, albeit at differing levels (Fig. 2C,D). No hybridization signal was observed in wood cells in the control sections hybridized with the *PdMYB221* sense probe (Fig. 2B). The similar result was obtained with the *PdMYB156* sense probe (data not shown). The preferential expression in developing secondary xylem (i.e. wood-forming tissues) and similar expression patterns of the two genes suggest that they may have similar functions in wood formation. It appeared that *PdMYB221* was more highly expressed than *PdMYB156* in the woody tissues. Therefore, *PdMYB221* was selected for further functional characterization in this study.

### PdMYB221 localizes to the nucleus and is a transcriptional repressor

**Figure 3:**
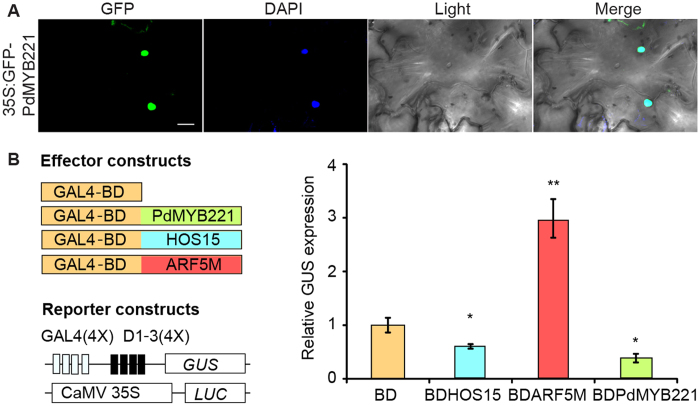
Subcellular localization and transcriptional activity analysis of PdMYB221. (**A**) Confocal images of localization of GFP-PdMYB221 in tobacco leaf epidermal cells. DAPI, a nuclear staining dye. Scale bars, 10 μm. (**B**) The transcriptional activity assays in Arabidopsis leaf protoplasts. (4X)-D1-3(4X), four upstream GAL4 DNA-binding sites in the promoter of GUS; CaMV 35S, a promoter of LUC reporter (the internal control). HOS15, a transcription suppressor, and ARF5M, an activator, were used as the controls. Student’s *t*-test shows the statistical significance, ^*^P < 0.05; ^**^P < 0.01.

The subcellular localization of PdMYB221 was examined using a tobacco leaf transient expression system^23^. Figure 3A showed that the PdMYB221:GFP fusion protein was colocalized to DAPI-staining nuclei, indicating that *PdMYB221* encodes a nuclear-localized protein. Previous study showed that Arabidopsis MYB4 acts as a transcriptional repressor^17^. We examined whether poplar PdMYB221 functions as transcriptional repressor using the GAL4-DNA-binding-domain (GAL4BD) and its binding sites (GAL4(4X)-D1-3(4X)-GUS)-based protoplast transient expression system^24^. Figure 3B indicated that PdMYB221 caused repression of the reporter gene expression by a similar amount (≈50%) conferred by a known repressor protein, *HOS15*^24^, compared with BD alone (set to 1). The negative control, ARF5M, activated GUS expression 3.1-fold compared with BD only. Therefore, PdMYB221 has the ability to repress the transcription of its target genes.

### Overexpression of *PdMYB221* affects secondary cell wall development in the Arabidopsis stems

**Figure 4:**
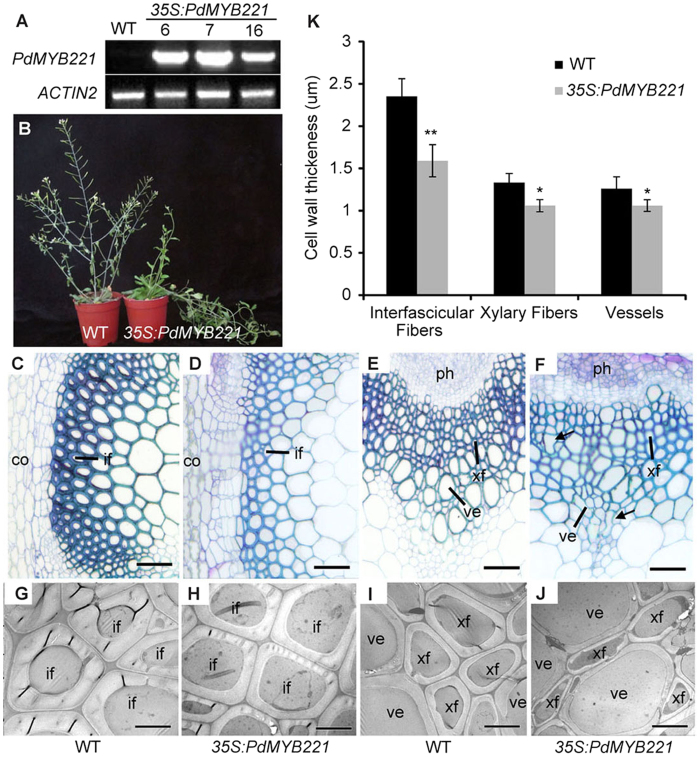
Reduction of secondary wall thickening in fibers and vessels by overexpression of *PdMYB221* in Arabidopsis. (**A**) RT-PCR analysis of *PdMYB221* expression (32 cycles) in three representative *PdMYB221* overexpression Arabidopsis lines. *ACTIN2* (26 cycles) was used as a control. (**B**) Six-week-old wild- type and *PdMYB221* overexpression (*35S:PdMYB221*) plants. (**C–J**) Cross sections of basal stems from 8-week-old wild-type (**C,E,G and I**) and *35S:PdMYB221* (**D,F,H and J**) plants. Interfascicular fibers (**C,D,G and H**); xylary fibers and vessels (**E,F,I and J**). (**K**) Cell wall thickness of vessels and fibers in the stems of wild-type and *PdMYB221* overexpression plants. Wall thickness was measured from transmission electron micrographs of fibers and vessels. Data are mean ± SD from 30 cells. Statistical significance, ^*^P < 0.05; ^**^P < 0.01. co, cortex; if, interfascicular fiber; ph, phloem; ve, vessel; xf, xylary fiber. Bars = 50 μm (**C–H**), or 5 μm (**G–J**).

In order to gain insights into the biological roles of *PdMYB221*, the *PdMYB221* gene was overexpressed under the control of the 35S promoter in wild-type Arabidopsis. At least 27 T_1_ transgenic plants were generated and confirmed by RT-PCR (Fig. 4A). Phenotypic analyses were carried out on homozygous T_3_ plants recovered from three independent transformants with high transcriptional levels. Compared with the wild type, transgenic lines overexpressing *PdMYB221* had little difference before four weeks, but were no longer able to remain upright when they reached heights of >15 cm, possibly because of the loss of secondary walls in the cells of the stems (Fig. 4B). Observation of cross sections of the basal inflorescence stems of six-week-old plants revealed that the cell wall thicknesses of fibers and vessels were thinner in the transgenic plants than in the wild type (Fig. 4C–J). Quantitative analysis showed that the cell-wall thicknesses of interfascicular fibers, xylary fibers, and vessels were decreased by 32%, 16% and 15%, respectively, in the transgenic lines, compared with the wild type (Fig. 4K). It is notable that *PdMYB221* overexpression lines were often found to have partially deformed vessels, probably because of the weakening of the vessels’ secondary walls (Fig. 4F).

**Figure 5:**
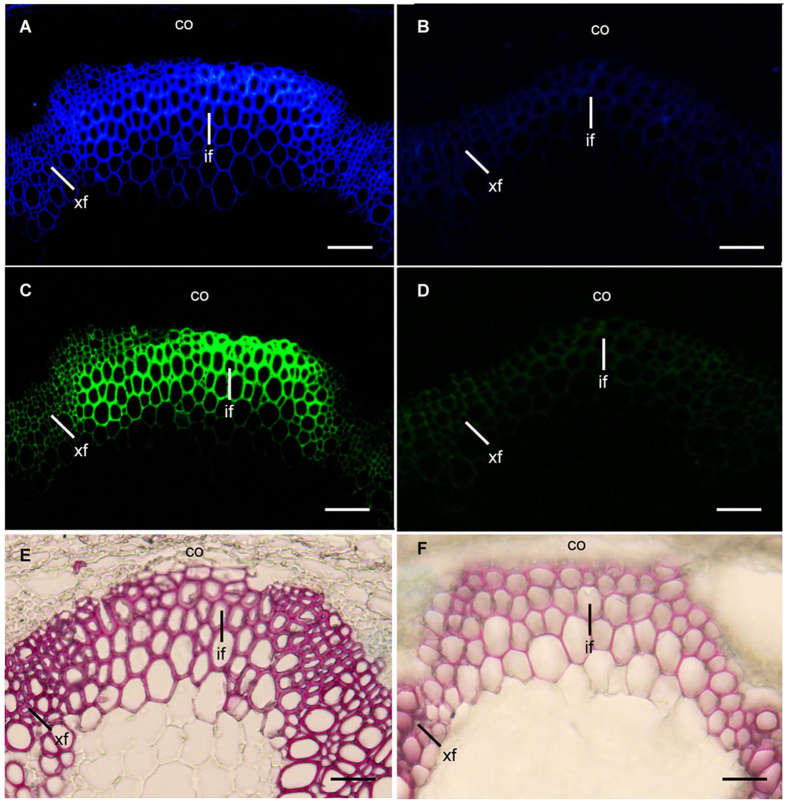
Deposition of cellulose, xylan and lignin in the stem secondary walls of *PdMYB221* overexpression plants. (**A,B**) Calcofluor White staining of stem sections showing little cellulose staining in the secondary walls of *35S:PdMYB221* (**B**) compared with the wild type (**A**). (**C,D**) Stem sections probed with the LM10 xylan monoclonal antibody showing few xylan staining in the secondary walls of *35S:PdMYB221* (**D**) compared with the wild type (**C**). (**E,F**) Phloroglucinol-HCl staining of stem sections showing few lignin staining in secondary walls of *35S:PdMYB221* (**F**) compared with the wild type (**E**). co, cortex; if, interfascicular fiber; xf, xylary fiber. Bar = 100 μm.

It is known that the change of wall thickness may reflect differences in cell-wall composition. We thus investigated the alteration of the cellulose, xylan and lignin contents in the stems of *PdMYB221* overexpression plants. Calcofluor staining of cellulose, immunolabeling of xylan with the monoclonal antibody LM10 and phloroglucinol-HCl staining of lignin revealed the presence of very weak signals in fiber and vessel cells of *PdMYB221* overexpression plants, while abundant cellulose, xylan and lignin were observed in those of the wild type (Fig. 5). Quantitative analysis of the cell-wall composition revealed that the amounts of cellulose, xylose and lignin were decreased by 10%, 26% and 28%, respectively, in the transgenic plants, compared with the wild type (Table 1). These results suggest that poplar *PdMYB221* may negatively regulate secondary cell wall biosynthesis in Arabidopsis.

### Overexpression of *PdMYB221* affects the expression of secondary wall biosynthetic genes and secondary wall-associated transcription factors in Arabidopsis

**Figure 6:**
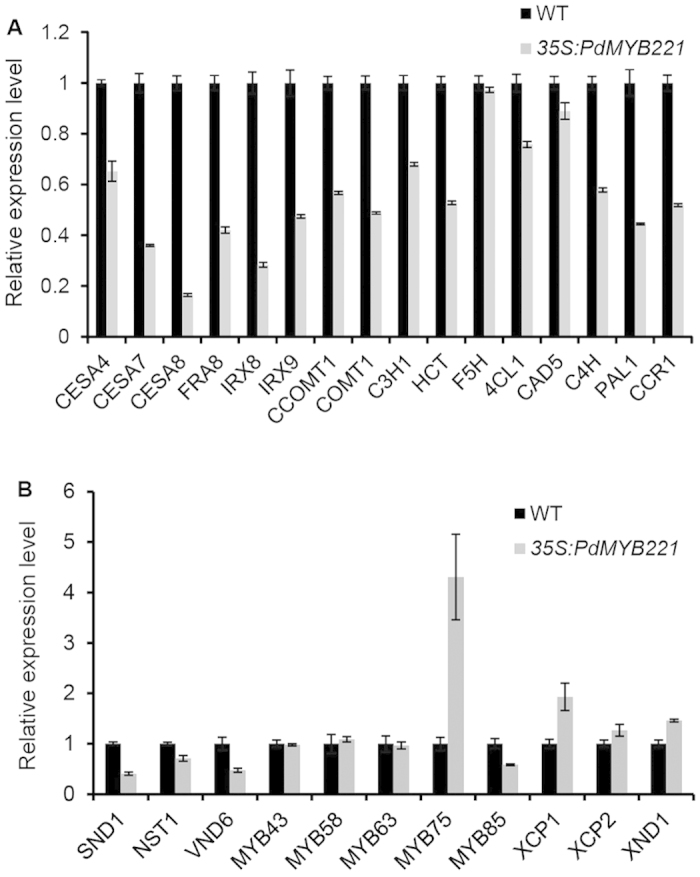
qRT-PCR detecting the expression of several genes associated with secondary wall biosythesis in the stems of wild-type and *PdMYB221* overexpression plants. (**A**) Genes involved in secondary wall biosynthesis. *CESA4*, *CESA7*, and *CESA8*, secondary wall-associated cellulose synthase genes; *FAR8*, *IRX8*, and *IRX9*, xylan synthase genes; *CCOMT1*, *COMT1*, *C3H1*, *HCT*, *F5H*, *4CL1*, *CAD5*, *C4H*, *PAL1*, and *CCR1*, lignin synthase genes. (**B**) Genes invovled in the regulation of secondary wall biosynthesis. The inflorescence stems of six-week-old plants were sampled. *ACTIN2* was used as an internal control. The expression level of each gene in the wild type was set to 1. Error bars represent SD of three biological replicates.

Given that overexpression of *PdMYB221* led to visible alteration in secondary cell wall formation in *Arabidopsis* stems, we used qRT-PCR to examine the expression of the related genes in *PdMYB221* overexpression lines. Expression of three cellulose synthetic genes (*CESA4*, *CESA7*, and *CESA8*), three xylan synthetic genes (*FAR8*, *IRX8* and *IRX9*), and nine lignin synthetic genes (*CCOMT1*, *COMT1*, *C3H1*, *HTC*, *4CL1*, *CAD5*, *C4H*, *PAL1* and *CCR1*) was suppressed in *PdMYB221* overexpression lines (Fig. 6A), consistent with the reduction of the cellulose, xylose, and lignin contents in transgenic plants (Table 1). This suggests that *PdMYB221* may negatively regulate secondary wall biosynthesis by repressing these secondary wall biosynthetic genes in Arabidopsis. Detection of the expression of eight secondary wall-associated transcription factors indicated that *SND1*, *NST1*, *VND6*, and *MYB85* were suppressed while *MYB75* was induced in *PdMYB221* overexpression lines (Fig. 6B). However, the expression of *MYB43*, *MYB58*, and *MYB63* in transgenic lines did not differ from the expression levels detected for these genes in the wild type. Interestingly, the expression of three programmed cell death-associated genes *XCP1*, *XCP2* and *XND1* was found to be induced in *PdMYB221* overexpression lines, suggesting that *PdMYB221* may be involved in programmed cell death.

### The *PdCESA7/8*, *PdGT47C*, *PdCOMT2* and *PdCCR1* promoters are repressed by *PdMYB221* and *PdMYB221* can bind to some of these promoters *in vitro*

**Figure 7:**
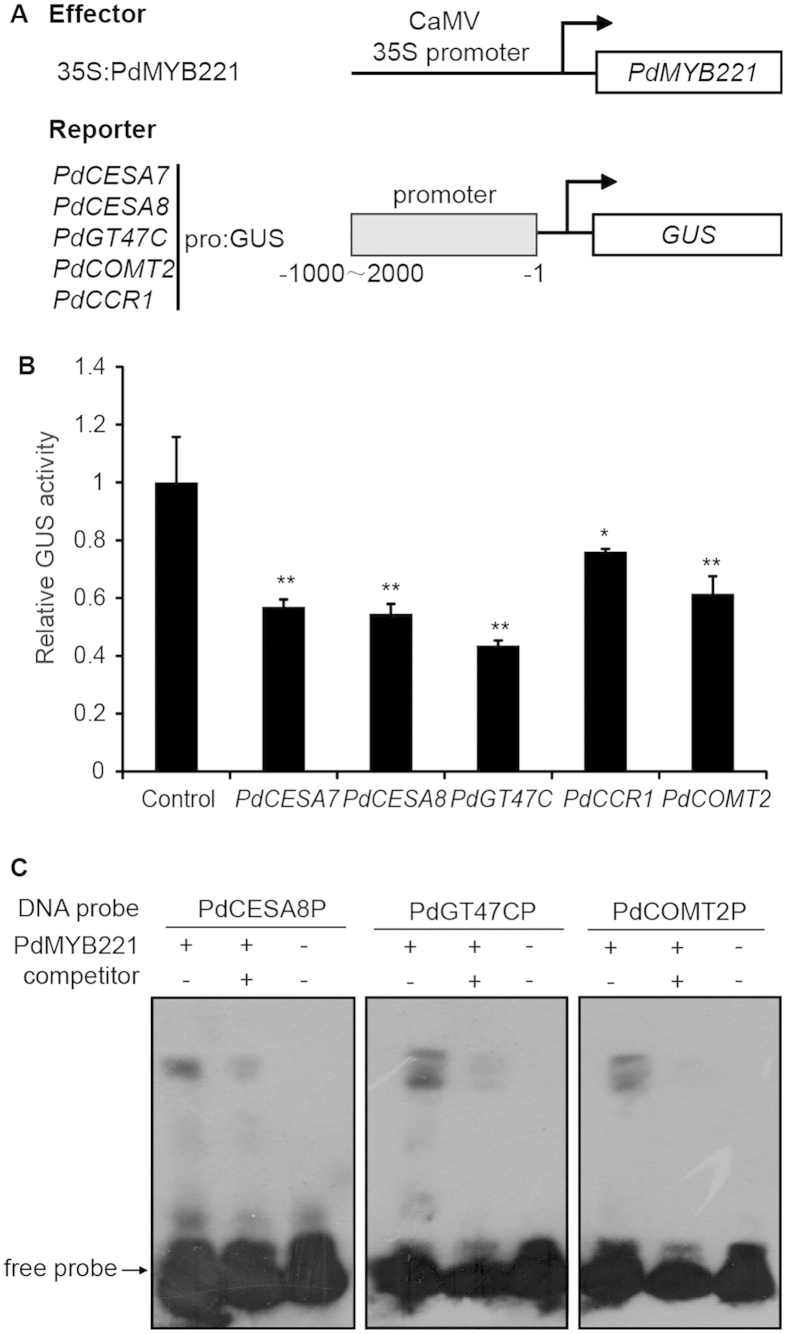
The *PdCESA7/8*, *PdGT47C*, *PdCOMT2* and *PdCCR1* promoters are repressed by *PdMYB221* and *PdMYB221* can bind some of these promoters. (**A**) Diagrams of the effector and reporter constructs used for transcription activity analysis. (**B**)Transcription activity analysis showing that *PdMYB221* represses the *PdCESA7/8*, *PdGT47C*, *PdCOMT2* and *PdCCR1* promoter-driven expression of the *GUS* reporter gene. The *GUS* expression in Arabidopsis leaf protoplasts transfected with no effector was used as a control and was set to 1. Error bars represent ± SD of three biological replicates. Statistical significance, ^*^P < 0.05; ^**^P < 0.01. (**C**) Electrophoretic mobility shift assays (EMSA) of PdMYB221 directly binding to the promoter sequences of *PdCESA8*, *PdGT47C* and *PdCOMT2*. PdMYB221 fused with HIS was incubated with biotin-labeled promoter fragments (located between −323 and −1 relative to the start codon) and subjected to EMSA by polyacrylamide gel electrophoresis. The biotin-labeled DNA fragments were detected with the chemiluminescence method. For competition analysis, unlabeled corresponding promoter fragments (competitors) in 30-fold (+) molar excess relative to the labeled probes were included in the reactions. Uncropped images of autoradiograms are shown in Supplementary Figure S1.

Since the expression of a subset of secondary wall biosynthetic genes was suppressed in *PdMYB221* overexpression plants, we employed transcriptional activation assays in Arabidopsis mesophyll protoplasts to investigate whether their poplar counterparts’ promoters could be repressed by PdMYB221. The *PdCESA7*, *PdCESA8*, *PdGT47C*, *PdCOMT2*, and *PdCCR1* promoters were amplified from poplar gDNAs. The five genes were selected, because their Arabidopsis counterparts *CESA7*, *CESA8*, *IRX8*, *COMT1* and *CCR1* had relatively lower expression than other genes in *PdMYB221* overexpression plants (Fig. 6A). The reporter and effector constructs were co-transfected into Arabidopsis leaf protoplasts, and subsequent assays of GUS activity in the transfected protoplasts showed that PdMYB221 was able to repress the expression of *PdCESA7*, *PdCESA8*, *PdGT47C*, *PdCOMT2*, and *PdCCR1* (Fig. 7A,B).

Analysis of the promoter sequences of these five genes showed that *PdCESA8*, *PdGT47C*, and *PdCOMT2* contained putative AC element consensus sequences ACC(A/T)A(C/A)C (Table S1), which have been shown to be the binding sites of lignin-specific regulatory genes^15^. We here used electrophoretic mobility shift assays (EMSA) to investigate whether PdMYB221 could bind directly to the *PdCESA8*, *PdGT47C*, and *PdCOMT2* promoters. Recombinant HIS-PdMYB221 fusion protein was expressed in *Escherichia coli* and purified for use in the EMSA. As shown in Fig. 7B, the recombinant PdMYB221 was able to bind the *PdCESA8*, *PdGT47C*, and *PdCOMT2* promoter fragments and cause mobility shifts. Addition of unlabeled *PdCESA8*, *PdGT47C*, or *PdCOMT2* promoter fragments competed with the binding in a dose-dependent manner, indicating that the binding of PdMYB221 to the *PdCESA8*, *PdGT47C*, and *PdCOMT2* promoters is specific. These results indicate that PdMYB221 has the capacity to directly suppress the expression of *PdCESA8*, *PdGT47C*, and *PdCOMT2 in vitro*.

## Discussion

Many transcription factors including R2R3-MYB family members have been shown to function as positive regulators of wood formation in tree species^5^,^6^,^7^,^8^,^25^. By contrast, only several negative regulators have been characterized in tree species. Of them, EgMYB1, an ortholog of Arabidopsis MYB46 in Eucalyptus, negatively regulates secondary cell wall formation in Arabidopsis and poplar^26^. Poplar PoptrKNAT7 can rescue the *knat7* phenotype when ectopically expressed in Arabidopsis, suggesting its potential negative role in secondary wall biosynthesis^27^. In this study, we provide evidence showing that poplar PdMYB221 may be involved in the negative regulation of secondary wall formation in Arabidopsis. The *PdMYB221* gene is predominantly expressed in fibers and vessels of poplar stems (Fig. 2), suggesting its potential role in wood formation. The preferential expression of *PdMYB221* in developing secondary xylem is consistent with its binding activity for the xylem expression-associated AC elements^28^. Transcriptional activity assays showed that *PdMYB221* suppressed *GUS* expression in a protoplast transient express system (Fig. 3B), indicating that it is a transcription repressor. Phenotypic analysis revealed that overexpression of *PdMYB221* decreased cell wall thickness of fibers and vessels in Arabidopsis inflorescence stems (Fig. 4). Consistent with this observation, the amounts of cellulose, xylose, and lignin were decreased in the stems of transgenic plants, compared with wild type (Table 1). This corresponded to the lower rate of cell wall polymer biosynthesis in transgenic lines. It is possible that the missing mass was not generated in transgenic lines. Alternatively, some intermediary compounds derived from cell wall polymer biosynthesis abnormally accumulated in *PdMYB221* overexpression cells, which would partially compensate for the loss of cell wall mass. Consistently, overexpression of switchgrass miR156 has been shown to accumulate more soluble compounds due to the significant decrease in cell wall mass^29^. The detailed mechanism needs to be investigated in future. Together, these results suggest that poplar PdMYB221 may be a negative regulator of secondary wall biosynthesis during wood formation. Given the fundamental importance of secondary walls in vascular plants, it is plausible that these plants contain MYB activators and repressors for fine-tuning regulation of secondary wall formation.

Detection of the transcriptional levels of secondary wall-associated genes using qRT-PCR showed that overexpression of *PdMYB221* in Arabidopsis suppressed the expression of fifteen secondary wall biosynthetic genes and four transcription factors (*SND1*, *NST1*, *VND6*, and *MYB85*) (Fig. 6). It is known that the four transcription factors positively regulate secondary wall biosynthesis^20^,^30^,^31^,^32^. It is possible that *PdMYB221* suppresses the expression of these secondary wall biosynthetic genes through the negative regulation of the four transcription factors. Further, SND1, NST1 or VND6 act as top master switches controlling secondary wall biosynthesis in Arabidopsis^20^,^30^,^31^, and *SND1* expression is feedback suppressed by its downstream *MYB4* gene^19^. Our results suggest that *PdMYB221*, an ortholog of *MYB4*, may suppress the expression of poplar orthologs of SND1, NST1 and VND6 through a similar negative feedback loop. Further experimental confirmation needs to be performed to better understand this. Our results also indicated that overexpression of *PdMYB221* in Arabidopsis induced the expression of *MYB75*, a negative regulator of secondary wall formation^33^. Since *PdMYB221* functions as a transcription repressor, one possible explanation for our result is that overexpression of *PdMYB221* may repress a repressor of *MYB75* in Arabidopsis. In addition to the indirect role of *PdMYB221* in controlling secondary wall biosynthesis, several lines of evidence showed that *PdMYB221* may directly suppress the expression of secondary wall biosynthetic genes in poplar. The *PdCESA8*, *PdGT47C* and *PdCOMT2* genes are the key genes synthesizing cellulose, xylan and lignin, respectively, in poplar. Their promoters contain the putative AC element sequences (Table S1). We used EMSA to show that the three promoters can be bound by PdMYB221 *in vitro* (Fig. 7B). Moreover, the *PdCESA8*, *PdGT47C* and *PdCOMT2* promoters can be suppressed by PdMYB221 in Arabidopsis mesophyll protoplasts (Fig. 7A). It should be noted that we cannot exclude the possibility that the poplar orthologs of other detected nine Arabidopsis lignin synthetic genes except for *F5H* may be also directly regulated by *PdMYB221*, because the expression of the nine genes was suppressed in *PdMYB221* overexpression plants (Fig. 6A) and the promoters of their poplar orthologs contain the AC elements (Table S1). Further studies are required to test this supposition. Our current results appear to be different from the finding of Jin *et al.* (2000). These authors showed that Arabidopsis AtMYB4, the ortholog of poplar PdMYB221, negatively regulates the accumulation of the UV-protectant compound sinapoylmalate, principally through suppressing *C4H* expression^17^. A possible reason for the difference of their lignin-associated targets is dose-dependent selection of target genes by *PdMYB221* and *AtMYB4*^17^.

**Figure 8:**
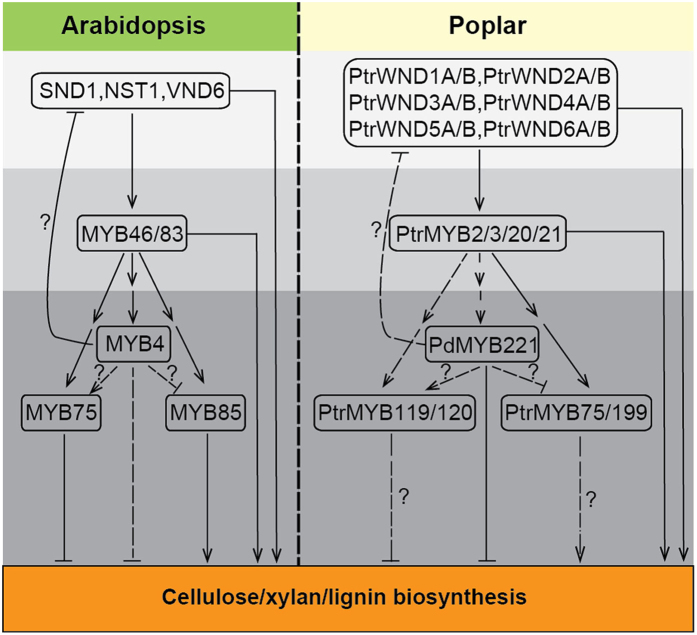
Diagram of the transcriptional networks regulating secondary wall biosynthesis in Arabidopsis and poplar. Arabidopsis NAC transcription factors (e.g., SND1, NST1, and VND6) function as the first-level master switches that directly activate the MYB46 and MYB83 targets^20^,^30^,^31^, which act as second-level master switches activating MYB4, MYB85 and MYB75 indirectly and in turn control secondary wall biosyntehsis^10^,^11^. Meanwhile, SND1, NST1, VND6, MYB46, and MYB83 have been shown to activate directly the expression of secondary wall biosynthetic genes. Further, *SND1* expression is feedback suppressed by the *MYB4* gene^19^. Poplar orthologs (PtrWND and PtrMYB) of these Arabidopsis regulators constitute a complicated network controlling secondary wall biosynthesis similar to that of Arabidopsis. Note that many additional AtMYB4/PdMYB221 -regulated transcription factors are not shown here. Arrows, positive regulation; line with block, negative regulation. Solid and dashed lines separately indicate known and non-verified targets. Question mark indicates a potential regulator relationship but unknown direct targets.

Our data on poplar PdMYB221, combined with other recent studies of poplar genes encoding orthologs of Arabidopsis NAC and MYB transcription factors in the secondary wall regulatory network support the idea that the transcription regulatory network governing secondary cell wall biosynthesis is largely conserved in poplar and Arabidopsis (Fig. 8).

In summary, this study demonstrated that poplar *PdMYB221* may be involved in the negative regulation of secondary wall biosynthesis through the direct and indirect suppression of the expression of key genes synthesizing cellulose, xylan and lignin. Our results mark another step toward the dissection of the molecular network that regulates secondary wall formation for genetic modification of poplar.

## Materials and Methods

### Plant materials and growth conditions

Poplar (*Populus deltoides* cv nanlin895) and Arabidopsis (*Arabidopsis thaliana*, Col-0) were grown in a greenhouse under standard conditions (temperature range 20–24 °C with a 16 h day from 06:00 h to 22:00 h and 55% relative humidity). For tissue-specific gene expression pattern analysis, leaves, roots, shoots, cortex, xylem and phloem were collected from 1.5-m-tall poplar plants. Samples were frozen immediately in liquid nitrogen and ground to powder in a cold mortar before subsequent RNA isolation.

### RT-PCR and quantitative RT-PCR (qRT-PCR)

Total RNA isolation and first strand cDNA synthesis were performed as described previously^34^. Semi-quantitative RT-PCR was performed with gene-specific primers (Table S2) to detect the expression of *PdMYB221* in wild-type and transgenic Arabidopsis plants. *ACTIN2* (At3G18780) was used as an internal control. qRT-PCR assays were conducted on a LightCycler^®^ 480 Detection System (Roche) with TransStart Green qPCR superMix (TransGen Biotech) following the manufacturer’s instructions. Baseline and threshold cycles (C_t_) were determined with the 2^nd^ maximum derivative method using LightCycler^®^ 480 Software release 1.5.0. Quantification of gene expression relative to the reference genes *ACTIN2* and *PdUBQ10* (BU879229) was determined using the 2^−ΔCT^ method^35^. Data presented represent the average of three biological replicates.

### *In situ* hybridization

The 144-bp 5′ UTR of *PdMYB156* and 141-bp 3′ UTR of *PdMYB221* were separately used for the synthesis of digoxigeninlabeled antisense and sense RNA probes with DIG RNA Labeling mix (Roche). The third internodes of the stems of 1.5-m poplar plants growing in a greenhouse were sectioned (8 mm thickness), fixed in 4% glutaraldehyde and embedded in paraffin for *in situ* mRNA localization according to our previous method^9^. Stem sections (10 μm) were cut on a rotary Leica RM 6025 microtome, mounted onto Superfrost Plus glass slides (Thermo Fisher, Waltham, USA) and hybridized with digoxygenin-labeled *PdMYB156* and *PdMYB221* antisense or sense RNA probes. The hybridization signals were detected by incubating with alkaline phosphatase -conjugated antibodies against digoxigenin and evaluating the subsequent color development with alkaline phosphatase substrates. The images were captured with an Olympus X51 light microscope and processed with Adobe Photoshop v7.0.

### Subcellular localization of PdMYB221

The *PdMYB221* coding region was amplified from stems of *P. deltoides* cv. nanlin895 and ligated between the CaMV 35S promoter and the 35S terminator in pK7FWG2 (Invitrogen). The resulting construct encoded fusion protein with PdMYB221 located at the C-terminus and GFP at the N-terminus. Infiltration of tobacco leaves with GFP-tagged PdMYB221 was performed as described previously^23^. After a 3-d post-infiltration period, transfected leaves were examined for green fluorescence signal using an Olympus FluoView FV1000 confocal microscope equipped with 488-nm argon laser.

### Overexpression of *PdMYB221* in Arabidopsis

The full-length *PdMYB221* cDNA was ligated downstream of the 35S promoter in the pEARLY100 vector (Invitrogen) to generate the overexpression construct. Five-week-old Arabidopsis plants were used for transformation via *Agrobacterium tumefaciens* (GV3101) following the floral dip method^36^. T_0_ transgenic plants were selected on basta (50 mg/L). At least three homologous T_3_ transgenic lines were used for examination of phenotypes.

### Microscopy and histochemistry

Basal stems (5–6 mm) of six-week-old Arabidopsis plants were fixed in 2.5% glutaraldehyde in phosphate buffer (pH 7.2), and embedded in resin (SPI-Chem.). Sections (1 μm thick) were cut with a Leica EM UC6 microtome and stained with 0.05% toluidine blue for light microscopy (Olympus DX51). For transmission electron microscopy, 70-nm-thick sections were cut with a microtome, stained with TBO, and observed using a Hitachi H-7650 electron microscope. Wall thickness was measured in metaxylem vessels and in interfascicular fibers next to the endodermis. At least three transgenic plants were examined.

Stems sections (1 μm thick) were stained for cellulose with 0.01% Calcofluor White and observed with a UV fluorescence microscope. Under the conditions used, only secondary walls exhibited brilliant fluorescence. Sections (50 μm thick) of stems were stained with phloroglucinol-HCl for lignin, which was shown as bright red color. For examination of xylan in secondary walls, 1-μm-thick sections were probed with LM10 monoclonal antibody, which binds to 4-*O*-methylglucuronoxylan^37^, and detected with fluorescein isothiocyanate-conjugated secondary antibodies. The fluorescence-labeled xylan signals were visualized with an Olympus DX51 light microscope.

### Analysis of cell wall composition

Inflorescence stems of six-week-old Arabidopsis plants were used as samples to analyze cell wall composition following the previous procedure^38^. Briefly, Alcohol insoluble residues (AIR) were prepared by treating the powder sequentially with 80% ethanol, 100% ethanol, and acetone. The resulting AIRs were dried under vacuum at 60 °C overnight and hydrolyzed for 2 h at 120 °C with 2 M trifluroacetic acid (TFA). The TFA-released materials were derivatized with 1-phenyl-3-methyl-5-pyrazolone (PMP) and analyzed on a Thermo ODS-2 C18 column (4.6 × 250 mm) connected to a Waters High Performance Liquid Chromatography (HPLC) system with a 2489 UV visible detector at a 245 nm wavelength. Elution solution A was 0.1 M phosphate buffered (pH = 7.0) and elution solution B was acetonitrile. The PMP derivative (10 μl) was injected, eluted at 1 ml/min with elution A: elution B = 82:18. To determine the cellulose content^39^, TFA-resistant materials were treated with Updegraff reagent (acetic acid/nitric acid/water, 8:1:2 v/v) at 100 °C for 30 min, and the resulting pellets were then completely hydrolyzed with 67% H_2_SO_4_ (v/v). The released glucose was measured using a glucose assay kit (Cayman Chemical) using a dehydration factor of 0.9. To determine the lignin content^40^, 3 mg of AIR samples was solubilized by acetyl bromide solution, and 2 M sodium hydroxide and 0.5 M hydroxylamine hydrochloride was then added to stop the reaction. Absorbance at 280 nm was measured using an UV-visible spectrophotometer (VARIAN Cary 50).

### Protoplast transformation and transient expression assays

The transcription activity assay was carried out in the transient-transformed protoplast^24^. The DNA binding domain (BD) from GAL4 was used in the system. The GAL4 BD-PdMYB221 fusion protein can bind to the GAL4 DNA-binding sites of the GUS reporter. Two known proteins HOS15, a transcription suppressor, and ARF5M, an activator, were used as the controls. The *GUS* reporter containing four upstream *GAL4* DNA-binding sites (*GAL4* (4X)-D1-3(4X)-*GUS*) and the luciferase (*LUC*) reporter were cotransformed with *GAL4 BD–PdMYB221* into Arabidopsis protoplasts. The *LUC* reporter carrying the luciferase gene under control of CaMV 35S promoter was used as the internal control to normalize the data for eliminating variations in the experiment.

For detecting the repressing activity of *PdMYB221* to five tested promoters, the promoters of *PdCESA7* (1948 bp), *PdCESA8* (1446 bp), *PdGT47C* (2021 bp), *PdCCR1* (1758 bp) and *PdCOMT2* (1219 bp) were individually ligated upstream of the *GUS* reporter after removing the 35S promoter in pBI221 to create the reporter constructs. The *PdMYB221* coding region was ligated between the 35S promoter and the NOS terminator after removing GUS from the pBI221 vector to create the effector construct. In each experiment, the expression level of the *GUS* reporter in the protoplasts transfected with the reporter construct alone was used as the control. The data presented are the means of three biological replications.

### Electrophoretic mobility shift assays (EMSA)

The PdMYB221 coding region was fused in frame with HIS in the pET28a vector and expressed in *Escherichia coli*. The recombinant protein was purified using Ni-NTA Spin Kit (QIAGEN, Germany). The *PdCESA8*, *PdGT47C*, and *PdCOMT2* promoter fragments used as the probes were amplified with primers labeled with biotin at the 5′ end (Table S2). The EMSA assay was conducted with a LightShift^®^ Chemiluminescent EMSA Kit (Thermo Fisher) according to the manufacturer’s instructions. Briefly, the labeled DNA fragments were incubated for 30 min with 100 ng of the recombinant protein in binding buffer (10 mM Tris, pH 7.5, 50 mM KCL, 1 mM DTT, 2.5% glycerol, 5 mM MgCl2, 0.05% Nonidet P-40 and 100 ng/μl poly (dl-dC)). The PdMYB221-bound DNA fragments were separated from the unbound fragments by polyacrylamide gel electrophoresis. The DNA was electroblotted onto a nitrocellulose membrane and detected by chemiluminescence.

### Statistical analysis

The data in the experiments of measurement of cell wall thickness and composition were subjected to statistical analysis using the Student’s *t* test. The quantitative differences between two groups of data for comparison in all these experiments were shown to be statistically significant (^*^P < 0.05; ^**^P < 0.01).

## Additional Information

**How to cite this article**: Tang, X. *et al.* Poplar PdMYB221 is involved in the direct and indirect regulation of secondary wall biosynthesis during wood formation. *Sci. Rep.* **5**, 12240; doi: 10.1038/srep12240 (2015).

## Supplementary Material

Supplementary Information

## Figures and Tables

**Table 1:** Cell wall composition analysis of the stems of wild-type and *PdMYB221* overexpression Arabidopsis plants.

**Composition**	**Wild type**	***35S:PdMYB221***
Man	13.3 ± 1.5	9.1 ± 1.1
Rha	6.4 ± 0.5	6.0 ± 0.3
Glc	14.5 ± 0.7	7.4 ± 0.6*
Gal	12.2 ± 1.0	10.3 ± 0.9
Xyl	75.4 ± 5.1	55.6 ± 3.6*
Ara	8.3 ± 0.1	9.2 ± 0.9
Fuc	0.9 ± 0.1	0.9 ± 0.3
Cellulose	214.4 ± 14.3	193.6 ± 15.2*
Lignin	291.6 ± 22.1	210.4 ± 25.6*

Alcohol insoluble residues (AIR) were prepared from the stems of six-week-old plants and determined by HPLC. The results are given as means ± SD (mg g^−1^ AIR) of three independent assays. Asterisks indicate significant differences (*t* test at *P* < 0.05) when compared with the wild type (*n* = 3).
