# Supplementary material for: Poplar PdMYB221 is involved in the direct and indirect regulation of secondary wall biosynthesis during wood formation
Source: Sci Rep. 2015 Jul 16;5:12240. doi: 10.1038/srep12240 (PMC4503951; doi:10.1038/srep12240)
Supplement: Supplementary Information [file srep12240-s1.doc]

**Poplar PdMYB221 is involved in the direct and indirect regulation of secondary wall biosynthesis during wood formation**

**Xianfeng Tang^1^, Yamei Zhuang^1, 2^, Guang Qi^1^, Dian Wang^1^, Huanhuan Liu^1^, Kairong Wang^2^, Guohua Chai^1^*, Gongke Zhou^1^***

^1^Key Laboratory of Biofuels, Chinese Academy of Sciences, Shandong Provincial Key Laboratory of Energy Genetics, Qingdao Institute of Bioenergy and Bioprocess Technology, Chinese Academy of Sciences, Qingdao, 266101, China

^2^Qingdao Engineering Research Center for Rural Environment, College of Resources and Environment, Qingdao Agricultural University, Qingdao, 266109, China

**Corresponding author:**

Guohua Chai (chaigh@qibebt.ac.cn);

Gongke Zhou (zhougk@qibebt.ac.cn)


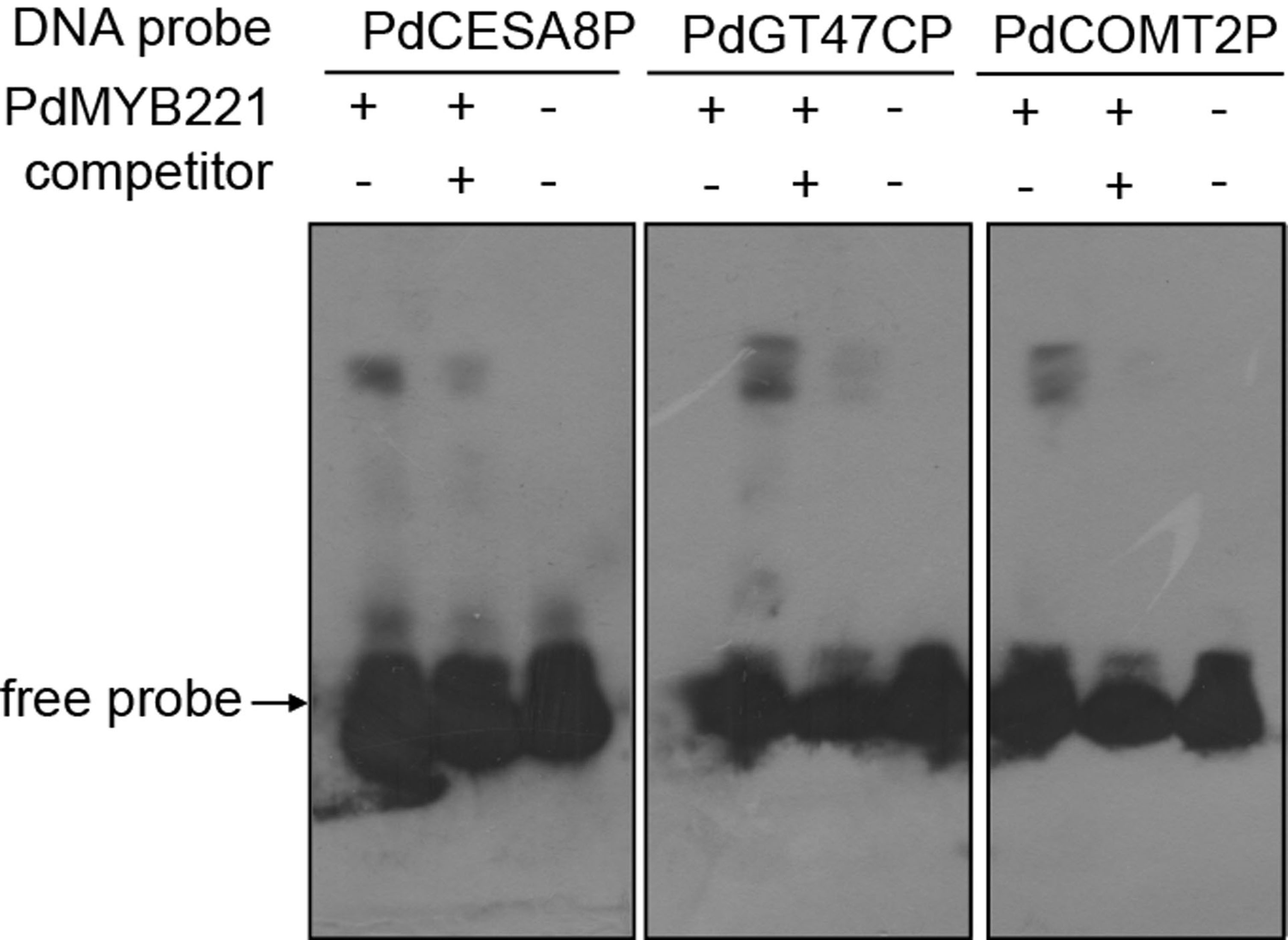


**Figure S1** EMSA analysis of PdMYB221 directly binding to the promoter sequences of *PdCESA8*, *PdGT47C* and *PdCOMT2*. These are uncropped images of the autoradiograms shown in Figure 7C.

**Table S1** The putative AC elements in the promoters of poplar *PdCESA8*, *PdGT47C*, *PdCOMT2* and other lignin synthetic genes.

| **Gene** | **Promoter sequence** |
| --- | --- |
| *PdCESA8* (Potri.004G059600) | ttttacagctaaaacatcaaagactcctggagagcatgccttaattctgttgcaggagaagattgtttcctgtttgtctcagtgttgcgtcaattgcttggttatccaagggctgccttttacagtttcatcattttgaccgaggtaaagtgcatgatacaccagccacccctttcaaggcacaccggcaaaccccatgatttttagagttttaagcttgtataagttgagtcacaggttgatgaacagctgtacaaaatggaattattatgtttcataggaatttcttcgctgttatatttctcagaatgattatcaaactgaatggatatcgttgactaaatttctgcaacaagtctcattacagttttgatggtttctatgagttggagcaaaatttgcatgatgtgaattggcttgtgatttatcaaattagaaagagtatgtatcaaaaggaatttatgagtttaatattcatg**accaaac**tttacttcccccatccccagaaaatacaaagatggctgaataagcaacgcttctgttgttatcgtccaattttaattttcatcgaataaattcagtcaggcaccaaaaagaacatgtcactcaattctttcatgaaatgcccttttttttttaatcatttttttgatgtctttttgatggttcgttgaatggctctgtttgcatctggtgttgcaatttcccgtgtagatttaacaggaaaatggattcttgagtcatttgcatcttcatcaaaagcaaggatctaagatctacgaaaatggatgcccatcatctgttcttggggataacactgatgtgaagtcgcaaagaataaatggttaatctgctaccattactgg**accaac**cagatatatggtctcagcgacataaaaagagatgtgctttatttttcatttttttcttttccttgagagacttaccctgatacatcgatatgatgattatctgaagcatgccgatgggagacgccagtacactgtttggatgctgccagttttcggtctacatggcggcccagccccggatctcgtgtttatattcaagagaggggacttgcattgaaagtaagtttgttaaagatgtgctctgcctaaca**gacaccttccttaaacttcccgcaaaaatttcaaaacttttctctcaccccaaaaattagacgttgcttctcatctctctctttctctgtaaacacatgacaccaactcctcaaccgccatgccccaccaacctcaa**ccctctcttctgcattaaaaaatgtcagcaccaccctctgagagacaagcaagacaccctagctttggttagggtctttgatataaaaaccaggccaaaatacaagattgagaagaagagggaagccctcttgaaagaactgaggaattgggggtgttaagagccggtgaagtggtaatcagcaaaATG |
| *PdGT47C* (Potri.009G006500) | aaacaaagataagggccagaatgatgatattattgaagatagatatatcgaatttctgtagcttgcgcaaccaaaattgaaaaaaaaaaagatattatttttttatacttacaacagtttaaatttaaaaattctttatcgactcgaattctaaattaaaaaaaaaaaccaagttaatcatagttaattcgtcaaactcgcgtcatggattatgaacttcattgggctagattgattttttattttattttatgataaaaaattcaaaaatctatttaccatctacccactattaaatgataaaattcaataaaaactatacactacaaaataaaattaaaaaaaacatattacaataaagcctagacgcgtgggttcgagctgcatgcacgccttatctaaattttttatttttaatttgattttttttattgtaaaaaacaatagccagcccgaatgaaaatgagaaccttaaactaaaatacacataaagataccatcaaactacaaaaagttgatgtgatgaaatgaaatcagagttgaaaaatataaaaagatggcctttggtgaagcaagttgatggcaggctaattttcaagtctttacacataaaaaccccaaacatcttcttgccgtgcacgggactttcataagaaagaccaggtcaggctaagtgtcattaaattttaattttaaaaatataatatgtttaaattacaccactattttgaagatttctgtaataattgatttttccagataagtacaagggtgatttcaggttgagaatctgtataaaaaaccataattatacctttgaaatgaattgattaaacaggaaattaaaaagtaagctcagttgggaataagaatttgtaaattatatactcggatattaaaacaaaaaagaaagcagatcttgtctttagaatttagatcgtccaaagtcaacaaggtatgatttccttcggcagcagcatctatgagttgagggagcaagttttatcccgtgatttcttcattgattctttttttcttccttttaattcacctataaaaatatttccgtttttttattttatttttttatttccgttcttatatttaacaaaaaaacgaaagtgataaccaactgcccattaattaagtctatcgataaaatgaagtgcatggagttactggtttccatattttagtggatctccatggacttggacccaccttgtttggtaattaacccttcattaattaactcatgcttaagacaagctaatctagcaatacgaggtgctcagtccctttttacccttcattaagacagcatgcagttttagatctccagccagtttgacctggaatctaggtaatttgaagtataagccagattgtattttaataataaaaaaaaaaaattaatttggtatgatccgtttaacccgacagcctaattaaaatccgtgtattttttttcttaaatgatattatttttatttttttgtaaaataaaaaaataaacccgctgtctaagatctagtatgaattgaaaactacgataaaagatcatgaaaattcttgaggctgtttcagtcaatcgagttttcaaaatgtcgtatggcagaccaggacaagcatagagaccgagacctccgccttgtttctttctcctccatcaccaccatatcaccttcataacataattatcttcaccttcctttctgacagaaccagaa**ctcgaccaaacccaattaaaaattcaaacgattattagccccaagagaaaaaaaaattgatgcaaagaccgtatgcatcttgcaccttatgtcattgtttattgtaggtgtaaaaacgaaatgccaccaaacctc**aacacctacaatctctgtcccttaatctccacttcattaccccaattaccaagaatgaaaaccccaccacaactgcatcctaaaagccccctttgatgatcttccttaaagcttctgtttcatgccctcttatattttgtgggttttaaatgaaacATG |
| *PdCOMT2* (Potri.012G006400) | attacgttacatgatctattactaagtacccaagtctctacgtcaatgttcaattttcagcaggtggttctgttagaatgtcccatccaaaatatggattcattgatacgatttttaagtccaaacaaccctcatattaagcaaaaccctcatattaaacaaaagattattattattattattattattgtttttgttgttgtgcttcttctttttctcgatcaacaaaatttttaccaacttcaagattttttttttatggttaaaggtatactaatatg**acctaat**aacttagaagtgtggattatagataaaattagcaattcgtgctatatagtgggttggatatttatttatataaaaaaattatatatataagtttttttttatgcatacttgtacaaaaaaaaaatataaatacaaatcaaatatttattcaatcaaatgataatagaaccagatatatatgaaattgattaaaaaaaatatatcatgttaggtcaacatattagaaatactatacaaaaataaatatttatatgtatataacacatacaaagattttctatagcgtgtgtttattcagtgagtttcatttatattaactttaaaatcattagttttataggatgtaaatttatcttttattaattttaaatgtgttcaataaatacaatcgggtgaatgtatcattatgtgattgaatatcttaatctgcatttatctcttaattttttcagttttttttttgttattgttaatgaatttttttttatttatataaatgattattgatttatttaattagatgctttatactttaattttttatatataaaaaaacatattaaaacaatctatatacctgatatttttatttttaaaaattataacccatgataaagaagttttat**aaacctacctgcttgacatattacatcatgttccaatagtctcccctgaaacaggttaaaaaaaaaaaagtttggcaaataagacgaggaaaaatatatagaagaaaaggtagggagtcagttctaggaagaagacatttgggcatcaagtagagagtagggaccaaccacaa**ggtggttgagcacttcaccatatatagcaccactttgcaacctctttttcagtattctcatatcctcttcacttcttttcttttcaccttcttcaaccttttgtttccttaaagaattcaatcttgatcaagATG |
| *Pd4CL1* (Potri.018G094200) | caatataatgagttggagtcgtactatttgatttaaaacacacgacttctaatttatgtttttttttctcaaaaaaaggaatggttggaaaaaaaagatgagctctgggattttttatatccacctgcatgatatttttattgaattgtatgattcagttttgagctcaacatgagattcttttaagcccgccagcttgattttttttatattttttgtttttttgttgtttttaattctattatttagtgtgagatttttattaaattgtatcattcaatattgagctcagcatgtattttcttagatccacctgcttgactttttttatatcttttattttttttgttgtcaattctattatttagcgtaagatttttattgaattgtatcattcagtattgagctcagcatggacttctttaagtccttctacttgatttttttatattttttatttttttattgtcaattctatcatttagtatgagatttttattgaattgtatcattcagtattgagttgatagtaaactaatcttaatatttccaattatataataaaataaaaaattaatttgacccagttgctggttgatatactgactaaatatcaattgaataattgaatagagatttttgttttatcaagtttaataacattactagaaactttttaaccatctaatttttttttttgggtgtctaacctaaaatgaaagcttgagccgataaacctatatatgtacacacacattttttctgtctcttttgtttgtatttgattgccttttatctttaccttttttctcggtggttagtttcttaaagaggggatatattgtggccatcgtaggaggtgagaggcggtgtctatcattaccagcacaacgaccacccatctccggctgattaaaggacaaggtggtggtaggtgttatcctacttggcatatgggtgttgtcttccagtcaaaaacgtaacatgcgcacgc**acctact**tcaccaccgatctccattctctactgttttttaaaagaattcaaattaggtgatcaaagattataataaaaaaacatcgttaaaatcctaccctaaaacacatggcaaccaatttattggagcccatatatatattcggatacgattagcatatcattaaataaaatctgctttcttggattgtgttagtgataaagagtgaaatccccatcaactccctctcaccggttctcctctatcatcacctccttatttattacctcaacctagaagcaaa**accaaac**acttacctttttgcttccaaaaaaagaagaaaaaaaaaatctctctctttccaaacaATG |
| *PdPAL3* (Potri.006G126800) | aaaataaaaatatcttattcacgataaagcatgatttacatagctaatttctatctaaacactggcaatcggagcctaatatgcatgtgaaataaaaataaatctaaaagtagatgttcaaagtgaatatggacctaaaaaaaggattttaatgacttcatgatcactactagactgtgaacctcttaattatatatttttttaattgataaaattgctgttaacaagccattcaaataaaaaattatgagaatattgagtgtttagtgacagcttgctattataataatagtgattattttttgaaataatttttatatagaaatatattaaaataatatttttttaattttttttaatatttacaaactaaaacaataaaaaaacattaaattaacacaaaaaagtaaaatttttaaaaacaccatttataccgaaaaacaaacatgcactaattcttttaagctaacttttgttcataaacacatgagctgaacatcccacgtcataattaatatatgatttgaaacaataaaaaggcaatgtgacgcaatttgttttttatagtttaacctggatgtccaggttagcttgcacgtacctcaactaatttcacgggcctccacagattatttgtttgagcttaactccattacatgtaagaattcagcaaaccaattatatacaataatttaactgttatgattttgagtttattttctaaaattttttgagtgtttttaagattactagagatttatatgattattaattttagtatccataaaattaattaaaatacaaataaattagcttgaaagattcatattaataataataataataaaaaagggccattttgggaaggtaaaagcatgattattaggtggatatatatatatatatatagaaaagaagggcaagacgaaatttcccatttctc**accaacc**acaacctcaccaccatgcatcacactaccacgatagtcaaatttacccttctacgccaatcaccaatatggatccacaaaaagagaccacgctccataatattgacccttgagattattcaagatcaatggccacaattgagtttcaacaaacccactttgtcccctcatgctt**acctaccaacc**tccatggctctatgcatattgcacagtattcaatactcccaacaatctattattctatttaaaccccctcccctccctctctcttctcctcaggaaatcccatcttccaaaaccaaagatttcctctactgcttctgctcttcctctcacggcaatcatttttctaacctcttcttctctctcatttacactttaatttcttctaacaaaacgaATG |
| *PdC4H*  (Potri.019G130700) | catttgcaatttgcacctccgcaaaacgttgatgacattcacagcatagcatgacaccagtcctttgaactaggatgataagcttgttccttaattaatttattcaaactagttagattttgtagcattgaaaataaataaattattttcttaattaatcggtattcctcaatttaataatttattttattaaaaaaacccatttcgccgttaacctttttttatttactctattctcttaataaaacaaaatacaaaatgaaatgaaaaacgggaaagtatttttaaatgctaaatgtatatatacccaataaaaatgtaagttaccaagtatatctaaaataataaattaaattaattaatttaatcataaacattaacccattaattgtattgatattagccatttaaattttatacactacaattaaaatttaattggctatatcaataaaatttattaattataaaaacacctataattaattataatttgagatcaatattctaacacgagggactattgacgaagtcccgattaatgaatattcttgaagtgtgtgtatatatacatatatgtatgtgtaaaacctgttggcataaaataaaacccgagttatgtatgaagaggaattctttgatttttgttaaaggatcaaaataatattgatttgaaaaaaaattaaaaagaatagtgaatggaataagtggagatcattttggattcttgaatgtgttagttagtttttctatttttttaacttaaatcaaggttttaaattaattttaaaaaatataatttaattggtcagattctgaattcagtttttaaagatcatcaatttaaatgctataaaatttaagattattaaaaatttacataaaaattaactttaaaattttaaaaaattaatcaagaaatataaaaattaactcagaaactacggtaaataaaaataaaaataataaggtcttgtattgagatgaagagagtcgcgcttgtatgcgtatatatataaggaatttgtgacatcatgtgcctcacctactacctctacaacgatcaatgctgtgttgtccctctcctatcacacctcagaactaacgccgttaactctgtttcagaa**acctaac**tggaagccacacgtcc**acctaac**tcatgaactatctttatctccaaccttccagttgctctttaattcttctactgcttataaaaccccctcccaacccccactcactcccc**accaacc**acctagtctctcctcctcttaattatattttcttcaatctaccactccttcttctccccagaaatctctttcagtactcctttggataaagaagatcatattccagtataATG |
| *PdHCT* (Potri.001G042900) | agtatacgtgagtctagtgagatgtcaaactcaacattcttgagtttagctatacattgaacccaagtatatgtgggtttggtgaaatgccag**acctaac**attcttagattcagttacgtgctgagccgtttgtgttgagcccaaatacacttgagtccacacgatatgctagaccccacacctttgagtttaattacatgccaaacctaagtacatgcagggttgtcaagatgctag**acctaac**acccttgaatgagtatcaattggttttgctccgtcattttgagccattgtggtctttctgaacccaagtacatgtaagtttagcaagctgtcatacccattcctcttgggtttaaaatcattttaggtctaaatatatacgatgttgataatattattaggcttcatattagatcaatagaatttgcttatttatttttatgtcttttaacatttattaatgccgtgtaagagatatttgtctctcattaatactatcaataaaaactaacgtttcaacctctctattcaaacaacatgacaaaattcaaatgttatatatattttttaaaccactaagataaatcattcatactttttatattatttaaatatttatactctatttttttagagcatttattatacatagcttagaagaaacaatctattttgaaatttaaaactcattataaaatcacagactttattattaaaaaggctttaaattccataacaaataattattttatagatgtgaagtcctctacaacctattatcaactacatgaaccacggaatgaaaaaaaaaacataaacatttgatcattctttctcccagcactcaaaacactcattcatgaatatacgggctcactgtctcgttatagctgcatcgtataattgatggaatttaaagatttaaaactagattttattacttctcgggaagtaatatcatgcctataatggttaaaatcagtttggcatgataaattgataaagaagattttaaattgaaataagatattaatttaataaataaataattaaaaaaacacttttaaaatattatttttaaaagataatcgatcccataacttgctagctaccctaataagtcacccctcccatgaagcccaggggacaagtatcctgcaagtcgcaccgagttgcttgcagagtggtgctc**accaacc**tggtctactttccaaagcactagaaaataataatttctataggcagcaaggttggtaacgcagggtgagctgattgaccggtggcatcgctgttggttgacgtggcaacggtgaaatgatatgctttgaaaagattgcttttaaaagtagtttttgttttaaaatgtattaatatatatattttttatttttttttaatatttaaaataacgaaatattggttcaaccacgctaaacaagccagaataatcaacaccattaggcgggggttggtggaaactaatagtttttgcttcccttgagaaacaggcaccttacacttcaggatcactattataaggaggcccttctacgtccttacaccaactccatttccaagttttcaattccatcgacacctgttcacatgctcttgtcttagattttatctctccttttctgcttcctagcctctcaaaggtataacttggtctgggagtgggatagatagagcattagggcggagaaagATG |
| *PdCCoAOMT1*  (Potri.009G099800) | aaaggtcacgcgggcattcctagcttctctataggcattttgctttgagcctccgaccattcccccctgctcttttcttattcacgatagaagtctgttcagttaaagaaaacatggcctgttttcctagaaatttatctataaaaaaggaaattcttgttaaaataaagaaaaaaattcaattgaaatacacttcccagctttcgaatgtttctattatattttgaaaaacatgtgaaaaatattttattaatgttcacttgaaaaaatggttgtggttgcttttcaaagtgttttttacttggaaatgctttaaaataatatttttttatttttaaaaaatcatttgtgatatcagcgcatcaaaatgatttgaaaacattaaaaaatattaatttaaaacaaaaaaataaaataaaataatttaaatttgtttaaatacttttaaaacataaaaaaaaaacagaatagaatttagacataaaaagtgttattctcgtcgtttaatagcgggaggtggtaagaaacatgctaagcattcacagttttggatattgattatccatgtgttaccctaaaaactgcctttttccatttcatgaaatcctcatttaatagcaattctttaaaggaggagactagagacagaggtggctgctgtcaacctagtcggtgaatttaaacttcaaccggcacatatatgcataccaagtataccctttacatctgccctaattaagactgtaaaacggatttggatttttgccgacaaaggctagtttgtggagaaaaaacaacgaagataaatttttagatgacaaagtcaacaatagttcgagagattctttaaaggactcatccgttgacggaggtggccatatgctaccaactcttggacgtggagtccctttggtaatttcacctatccctcacccaatttctattagcagttagcacatgtaatttatgattggtgagcccagcacaaatcttttccagttaaacacatatattaatttatgattaattatttaattctctccactcttaacaaattaatcatacatggcataacattttagcttttgatctcgagaatctct**acctaac**cattgacttctttactgttcaggaatcttagaaaaaaggaggacaaaaaaaaatatgcccaataaattatttaagaatttgaaccgatatttggtgtcatagatcccaaaaatgacgtcagcgatgcctcagggaaggagtaccactagcccacagcacgatacgatcaccaacaaggtgggtcccatatttggtgggccaaaaacccacattatccttcgtcctaactacaggaacctc**accaacc**ccctcccggttggtagccggtccagcctccccgctactccaattcaaaccgggctctcatttccaataaataccacccgccctttaccattttcgatcaggttaggcatcactaccatcatcaacaacaaaaataaaaattccaaggccaagaaagagatcgtagtttaattagaagatatacacaataATG |
| *PdC3H1*  (Potri.003G006000) | acactttaccatcctcacccattttttaccaaattcaccatccaaatatcatatatataaaatcttgtgttgttgttttttacttggtggatttgagaccacgttagagcccgatcttgctgagggtttagattatcgagttaatctgtggattaattttaaaataatattttttagatagtttttttttaataacattattttaaatataaattaaaaaaaattaaaactgattcaattaagtttcatttgagatttctttatccagtttgacttatcaagatacctaaatttaatacacaaattgacttgaaagcaaatctaatctaagttaggttttaagagtccatttagaaacatgtatccttaaattttgaaaaagaattattaaaaattattattttttgtactgatctcaaaaataatttttaaaaaataaaaaatattattttgatgtatttctaagctaaaaacacgttgaaccgcaatcaccattacaatttcaagcttaaaatacattaaaatgtgtgattgtgggagtttgggagtgtgattatggttacttttcaaagtatttttcacttggaaatacattaaaataatattttttaaaaaaaattatttttaatatcagcacattaaaaatatctaaaaacactaaaaaaatattgtttttatacaaaaaaataaaaaataaaaatatataatttaaatcgcattctcgaacattacatatctcccggtccagccgtcaaaagcaacttttaaattatataatagtagtagataacattatgtgccccgtaattataaatttctatagcaatttccctctcaattattacaaaccttttaacttatcttattttcttaattacaaatatgtgggctgcgagaaatgaatagaatggtctcccccttcaccaacttttgattcctccttctttgcttgttaaaaagtctcacttc**accaacc**accaaatactaaaaaaaacaaaaaaacaaaaaacccgctctactatcttttcaccgagagtgaagtcacgcgccaccgttcttgagcacgtgggacattgtaattggtcaatgtttccccacaatgggcattgacaagtgacacttttttcccctccctctcctatgacagcgactataagcagaagcaaagagtcgcacatcgtccctcaacttcttaaatttattcatgttagaccctcttctttaagtttgatcaaactcactacttcaatctcaatgaaatgcattcttacctcgtgaaaaaactcaattattaattaaatgacttgaatgaacatctgaaattggatttgtatgaaagtatgaaagtaaaaagggtttatttgacccaatttaaagtgagggattgagttggtaatttgtggaaattagagggaccatgaatgaacaaaatccactcaccaactccctccctcgcagagaccaactccattcctaactccattcctacaatttatattctgccaaggcccttcagaaaaattagaccctttctctcccttttttttttctctccaca ATG |
| *PdCAD5* (Potri.009G095800) | ttgattattatttattttttttagataatttatggaattatatttttttttcaatttcattctcatttaacttttttaatttgtaagatttgttcctcattattttaataaacttaaaaaaataaaacattaataaattatttttcagctcatttttcatgacataaccaaatactggaaattgttttctaacttatttttcattacactatcaaatatcggaaaataattcactttccaaaaggaaactacttttcagcaaacaaacggggcctaagtgtttttatttttacattgaaagtgtctaaatagatttgagctcgatcgggaagacttttggttttgagttgatttaggttttgaggcatttttttgtggtttttagaggccattgattagtctaataaggttcttctccataatttttctagcttatttatcattgtttaaatattaattttttattatcatatttggtgtgtttaattcatctataatttttttatatatattttacatatattaaacttactttaaaaattaaaaatgtttatttttttataatatttttaatatgtgcaaccttacaatatctttccttttattatattcaataaattttccacgtgaatcgatttttattataaaaatattttaataaacaaattcattatacataatcggataaattacccatattacgatattaaatattttgatttatatttgtttcttgttttgtttttatcataaatttattttttttattgaattatcttgaattgatgacgtaggaaacatgataaacatgtaatctagatatatctcatgtctaggtcatgggtttcacgtgttagttcagctttacccaaaataatttttttatttgttattattgttaccttattttttcatcatattattaaattaattaaaatttaatcaaaacattaattttttcttacttttttttaaaatataatcttctcttaatttttttttttcatgtttaaaaaaatttcagtcgacggcacaacaatccagtaaataccaagggtatattgtcgccactcaccaccaactacgtcaattaagcaaataatataattaggcaactgtgtaaccaccatggaaattaagatattcctttcatgaaatacttaattagtgacgtatacatgatgctccaaacctcatcacagattcagtgttcttaactattatgttcccttttgtttcccaagaaccatgagttaatcaggaccatcgatactactgaggccccaccaatgttttgatcatgtggacaatgttcacttgattttcaactttgaagaaatgacccatggttgtggaagcagaggatggcgccactccatcacatttc**acctacc**accacccgtaaaatatgcggagctgtccttgtcttttttgttgccaagtaacctttgccattctttattgtgcttttgtatatatactcatccatagtggcttataattcttcaactctccacagaaactccatctctctctcttagcctcattgtttcaagaaaATG |

Note: Underline shows the putative AC-element site. The bold fragments in *PdCESA8*, *PdGT47C*, and *PdCOMT2* promoters represent the labeled probe in the EMSA assay.

**Table S2** Primers used in this study

| **Gene Name** | **Accession number** | **Primers** | **Sequence(5'-3')** | **Goal** |
| --- | --- | --- | --- | --- |
| *PdMYB156* | Potri.009G134000 | PdMYB156realtimeF | CCAGCCTGATCATCACCAGCCA | For amplifying transcript of *PdMYB156* by RT-PCR or qRT-PCR |
|  |  | PdMYB156realtimeR | GTGCTGCCACTGCTCCCAACA |  |
|  |  | PdMYB156insituF | ACCGATTCGTTGTAGGAT | For amplifying 5' region of *PdMYB156* cDNA using for in situ hybridization |
|  |  | PdMYB156insituR | ATATTTATATCCCTATTGTTG |  |
| *PdMYB221* | Potri.004G174400 | PdMYB221realtimeF | TGCTTGCAGCTTGGGGCTACA | For amplifying transcript of *PdMYB221* by RT-PCR or qRT-PCR |
|  |  | PdMYB221realtimeR | AGCCAGTTTTGGAGCCAGCACT |  |
|  |  | PdMYB221insituF | ATTTGTACAGTTTAGTGATCAT | For amplifying 3' region of PdMYB221 cDNA using for in situ hybridization |
|  |  | PdMYB221insituR | GAACATCTGACATAGCCCAACTT |  |
|  |  | PdMYB221cDNAF | ATGGGAAGGTCTCCTTGCTGT | For amplifying *PdMYB221* full-length cDNA |
|  |  | PdMYB221cDNAR | TCATTTCATCTCCAAACCTCTAT |  |
|  |  | PdMYB221TAAF | GAATTC ATGGGAAGGTCTCCTTGCTGT | For amplifying *PdMYB221* full-length cDNA for transcription activation assay |
|  |  | PdMYB221TAAR | GGATCCTCATTTCATCTCCAAACCTCTAT |  |
| *PdUBQ10* | BU879229 | UBQ10F | GTTGATTTTTGCTGGGAAGC | For amplifying transcript of *PdUBQ10* by qRT-PCR |
|  |  | UBQ10R | GATCTTGGCCTTCACGTTGT |  |
| *ACTIN2* | AT3G18780 | ACTIN2RTF | GGTAACATTGTGCTCAGTGGTGG | For amplifying transcript of *ACTIN2* by RT-PCR or qRT-PCR |
|  |  | ACTIN2RTR | AACGACCTTAATCTTCATGCTGC |  |
| *CESA4* | AT5G44030 | CESA4realtimeF | GGATCAGCTCCGATCAATTT | For amplifying transcript of *CESA4* by qRT-PCR |
|  |  | CESA4realtimeR | ACCACAAAGGACAATGACGA |  |
| *CESA7* | AT5G17420 | CESA7realtimeF | CAGGCGTACTCACAAATGCT | For amplifying transcript of *CESA7* by qRT-PCR |
|  |  | CESA7realtimeR | TGTCAATGCCATCAAACCTT |  |
| *CESA8* | At4g18780 | CESA8realtimeF | ACGGAGAGTTCTTTGTGGCT | For amplifying transcript of *CESA8* by qRT-PCR |
|  |  | CESA8realtimeR | GGTCTGTGTTGGAACAATGG |  |
| *IRX8* | AT5G54690 | IRX8realtimeF | GTGGTCACAGGGAAAGGATT | For amplifying transcript of *IRX8* by qRT-PCR |
|  |  | IRX8realtimeR | AGCAAGAGAGGAGCAAGGAG |  |
| *IRX9* | At2g37090 | IRX9realtimeF | TTTGCGGGACTAAACAACAT | For amplifying transcript of *IRX9* by qRT-PCR |
|  |  | IRX9realtimeR | ATCGGAGGCTTTGTCTCTGT |  |
| *FRA8* | AT2G28110 | FRA8realtimeF | GACTTGTTGAATCGGTGGCTC | For amplifying transcript of *FRA8* by RT-PCR or qRT-PCR |
|  |  | FRA8realtimeR | GAAAGAGTTTGACCTTCTAAC |  |
| *4CL1* | AT1G51680 | 4CL1realtimeF | TCAACCCGGTGAGATTTGTA | For amplifying transcript of *4CL1* by qRT-PCR |
|  |  | 4CL1realtimeR | TCGTCATCGATCAATCCAAT |  |
| *C3H1* | AT2G40890 | C3H1realtimeF | GTTGGACTTGACCGGATCTT | For amplifying transcript of *C3H1* by qRT-PCR |
|  |  | C3H1realtimeR | ATTAGAGGCGTTGGAGGATG |  |
| *HCT* | AT5G48930 | HCTrealtimeF | GCCTGCACCAAGTATGAAGA | For amplifying transcript of *HCT* by qRT-PCR |
|  |  | HCTrealtimeR | GACAGTGTTCCCATCCTCCT |  |
| *F5H* | AT4G36220 | F5HrealtimeF | CTTCAACGTAGCGGATTTCA | For amplifying transcript of *F5H* by qRT-PCR |
|  |  | F5HrealtimeR | AGATCATTACGGGCCTTCAC |  |
| *CAD5* | AT4G34230 | CAD5realtimeF | TTGGCTGATTCGTTGGATTA | For amplifying transcript of *CAD5* by qRT-PCR |
|  |  | CAD51realtimeR | ATCACTTTCCTCCCAAGCAT |  |
| *C4H* | AT2G30490 | C4HrealtimeF | ACTGGCTTCAAGTCGGAGAT | For amplifying transcript of *C4H* by qRT-PCR |
|  |  | C4HrealtimeR | ACACGACGTTTCTCGTTCTG |  |
| *PAL1* | AT2G37040 | PAL1realtimeF | AAGATTGGAGCTTTCGAGGA | For amplifying transcript of *PAL1* by qRT-PCR |
|  |  | PAL1realtimeR | TCTGTTCCAAGCTCTTCCCT |  |
| *CCR1* | AT1G15950 | CCR1realtimeF | GTGCAAAGCAGATCTTCAGG | For amplifying transcript of *CCR1* by qRT-PCR |
|  |  | CCR1realtimeR | GCCGCAGCATTAATTACAAA |  |
| *COMT1* | AT5G54160 | COMT1realtimeF | TTCCATTGCTGCTCTTTGTC | For amplifying transcript of *COMT1* by qRT-PCR |
|  |  | COMT1realtimeR | CATGGTGATTGTGGAATGGT |  |
| *CCOMT1* | AT4G26220 | CCOMT1realtimeF | CTCAGGGAAGTGACAGCAAA | For amplifying transcript of *CCOMT1* by qRT-PCR |
|  |  | CCOMT1realtimeR | GTGGCGAGAAGAGAGTAGCC |  |
| *SND1* | AT1G32770 | SND1realtimeF | CAAGCTTGAGCCTTGGGATA | For amplifying transcript of *SND1* by qRT-PCR |
|  |  | SND1realtimeR | TGGTCCCGGTTGGATACTT |  |
| *NST1* | AT2G46770 | NST1realtimeF | TCATCCGACCGAGGAAGAGC | For amplifying transcript of *NST1* by qRT-PCR |
|  |  | NST1realtimeR | GAAGCTCCTCCGACGGGACT |  |
| *VND6* | AT5G62380 | VND6realtimeF | GCCATGGGACATCCAAGA | For amplifying transcript of *VND6* by qRT-PCR |
|  |  | VND6realtimeR | TGTGGCTAAAGAAATACCATTCC |  |
| *MYB43* | AT5G16600 | MYB43realtimeF | TTGACCTCAAGTGGCTTTCATCCGA | For amplifying transcript of *MYB43* by qRT-PCR |
|  |  | MYB43realtimeR | AGCTGCTCAAATCATTGATGTCCCA |  |
| *MYB58* | AT1G16490 | MYB58realtimeF | CCAGAGAACAGAGCTCTTCAAGAG | For amplifying transcript of *MYB58* by qRT-PCR |
|  |  | MYB58realtimeR | ATGTATGAGGAGCTCGTAACTCTC |  |
| *MYB63* | AT1G79180 | MYB63realtimeF | GAACAGCTCAGGCTCAAGAGCAAC | For amplifying transcript of *MYB63* by qRT-PCR |
|  |  | MYB63realtimeR | ATGTATCATGAGCTCGTAGTTCTT |  |
| *MYB75* | AT1G56650 | MYB75realtimeF | TGGCACCAAGTTCCTGTAAG | For amplifying transcript of *MYB75* by qRT-PCR |
|  |  | MYB75realtimeR | AAGCCTATGAAGGCGAAGAA |  |
| *MYB85* | AT4G22680 | MYB85realtimeF | TCGATCCTATGACCCATCAACCCCT | For amplifying transcript of *MYB85* by qRT-PCR |
|  |  | MYB85realtimeR | TGTTGTCGTGACACTTATCTCCACG |  |
| *XCP1* | AT4G35350 | XCP1realtimeF | TTGACCCATGAAGAGTTCAAAGGAAGA | For amplifying transcript of *XCP1* by qRT-PCR |
|  |  | XCP1realtimeR | GAAAGCGAACTCAGATTCCCTGTTG |  |
| *XCP2* | AT1G20850 | XCP2realtimeF | TTGCGAGATGCAAAAGGAT | For amplifying transcript of *XCP2* by qRT-PCR |
|  |  | XCP2realtimeR | GCCAATGCCTTCAAGAGACT |  |
| *XND1* | AT5G64530 | XND1realtimeF | ACCCTGATGTCATCCCCGACCTT | For amplifying transcript of *XND1* by qRT-PCR |
|  |  | XND1realtimeR | TGGCTCGTCCATTCCCATTGATCCC |  |
| *PdCESA7* | Potri.005G194200 | PdCESA7proF | TACCTGTGCCAAACTGCCATAAG | For amplifying *PdCESA7* promoter for transcriptional activity assay |
|  |  | PdCESA7proR | GTTGAGCAATGGAAAGGGGC |  |
| *PdCESA8* | Potri.004G059600 | PdCESA8proF | CCTGACATAAAGAACGGTAGCAAT | For amplifying *PdCESA8* promoter for transcriptional activity and EMSA assays |
|  |  | PdCESA8proR | TTTGCTGATTACCACTTCACCG |  |
| *PdGT47C* | Potri.009G006500 | PdGT47CproF | AAACAAAGATAAGGGCCAG | For amplifying *PdGT47C* promoter for transcriptional activity and EMSA assays |
|  |  | PdGT47CproR | GTTTCATTTAAAACCCACAAAATA |  |
| *PdCCR1* | Potri.001G046100 | PdCCR1proF | TAAATTGAAAAATATTTTTCAGT | For amplifying *PdCCR1* promoter for transcriptional activity assay |
|  |  | PdCCR1proR | GTTGTTTTGATATGTGTATTATTATG |  |
| *PdCOMT2* | Potri.012G006400 | PdCOMT2proF | ATTACGTTACATGATCTATTACTAAG | For amplifying *PdCOMT2* promoter for transcriptional activity and EMSA assays |
|  |  | PdCOMT2proR | CTTGATCAAGATTGAATTCT |  |
